# JWA down-regulates HER2 expression via c-Cbl and induces lapatinib resistance in human gastric cancer cells

**DOI:** 10.18632/oncotarget.12374

**Published:** 2016-09-30

**Authors:** Ling Ma, Weiyou Zhu, Qiang Wang, Fengming Yang, Jing Qian, Tongpeng Xu, Shouyu Wang, Jianwei Zhou, Yongqian Shu

**Affiliations:** ^1^ Department of Oncology, the First Affiliated Hospital of Nanjing Medical University, Nanjing, People's Republic of China; ^2^ Department of Molecular Cell Biology and Toxicology, Jiangsu Key Laboratory of Cancer Biomarkers, Prevention and Treatment, Cancer Center, Nanjing Medical University, Nanjing, People's Republic of China; ^3^ Jiangsu Key Laboratory of Cancer Biomarkers, Prevention and Treatment, Collaborative Innovation Center For Cancer Personalized Medicine, Nanjing Medical University, Nanjing, People's Republic of China

**Keywords:** gastric cancer, lapatinib, HER2, JWA, c-Cbl

## Abstract

Human epidermal growth factor receptor 2 (HER2) targeted therapy is currently considered as the standard treatment for HER2-positive advanced gastric cancer (GC). However, unsatisfactory results of recent phase III clinical trials involving lapatinib suggested biomarkers for selection of patients. The aim of this study was to identify JWA as a biomarker for lapatinib resistance in GC cells and elucidate the underlying mechanisms. Lapatinib was effective to the intrinsic cisplatin-resistant GC cells. JWA activation conferred lapatinib unresponsiveness, but reversed cisplatin resistance in GC cells. Whereas, deletion of JWA significantly restored lapatinib suppression on proliferation and lapatinib-induced apoptosis. JWA-induced down-regulation of HER2 and activation of ERK phosphorylation led to lapatinib resistance. Furthermore, c-Cbl represented a novel mechanism for HER2 degradation enhanced by JWA in GC cells. Taken together, JWA is a potential predictive marker for lapatinib resistance, targeting the patients that may benefit from lapatinib treatment in human GC.

## INTRODUCTION

Gastric cancer (GC) is one of the most common malignant cancers and the third most common cause of cancer-related death worldwide [1]. Overexpression of HER2 has been reported to be associated with poor prognosis in several human cancers, such as breast cancer and GC [2, 3]. HER2 targeted therapy has aroused great interest in the treatment of advanced GC [4].

Trastuzumab and lapatinib have been approved for the treatment of patients with advanced HER2 positive breast cancer [5–7]. Whereas, in heterogeneous advanced GC, lapatinib has not been widely acknowledged for clinical treatment so far. A serious of lapatinib-concerned clinical trials in advanced GC have been undergoing with great attentions [8–10]. Unfortunately, the disappointing results of the negative phase III TyTAN and LOGiC trials have been reported recently [9, 10]. The unsatisfactory outcomes have provoked fierce debates among academics. Yelena Y. Janjigian claimed that “The success of future trials examining molecular targets will depend on biomarker-driven patient selection” [11] (p. 402). In addition, a study revealed that MET, HER3, IGF1R, and INSR pathways activation may be responsible for lapatinib unresponsiveness in HER2 positive GC [12]. Thus, it is significant to identify biomarkers for the intrinsic resistance to lapatinib and for predicting which patients will benefit from lapatinib therapy.

The *JWA* gene, also called ADP ribosylation-like factor 6 interacting protein 5 (*ARL6ip5*), serves as a tumor suppressor gene in GC [13, 14]. JWA enhances the apoptosis of certain chemotherapeutic agents [15–17]. In resectable human GC, JWA is a novel candidate predictive factor and prognostic marker for benefits from adjuvant platinum-based chemotherapy (FLO or FLP) [13]. In addition, JWA may be a valuable target for reversal of cisplatin resistance in human GC by negatively regulating XRCC1 through the CK2/p-XRCC1 pathway [17].

The objectives of the present study were to investigate the roles of JWA in lapatinib resistance of GC cells and to elucidate the underlying mechanisms. Our results demonstrated that JWA negatively regulated HER2 through the JWA/c-Cbl/HER2 pathway in GC cells and promoted unresponsiveness to lapatinib. Besides, JWA-induced activation of MAPK pathway contributed to lapatinib resistance. JWA could be a potential predictive marker for lapatinib unresponsiveness in human GC.

## RESULTS

### Cisplatin-resistant GC cells sensitize to lapatinib

**Figure 1:**
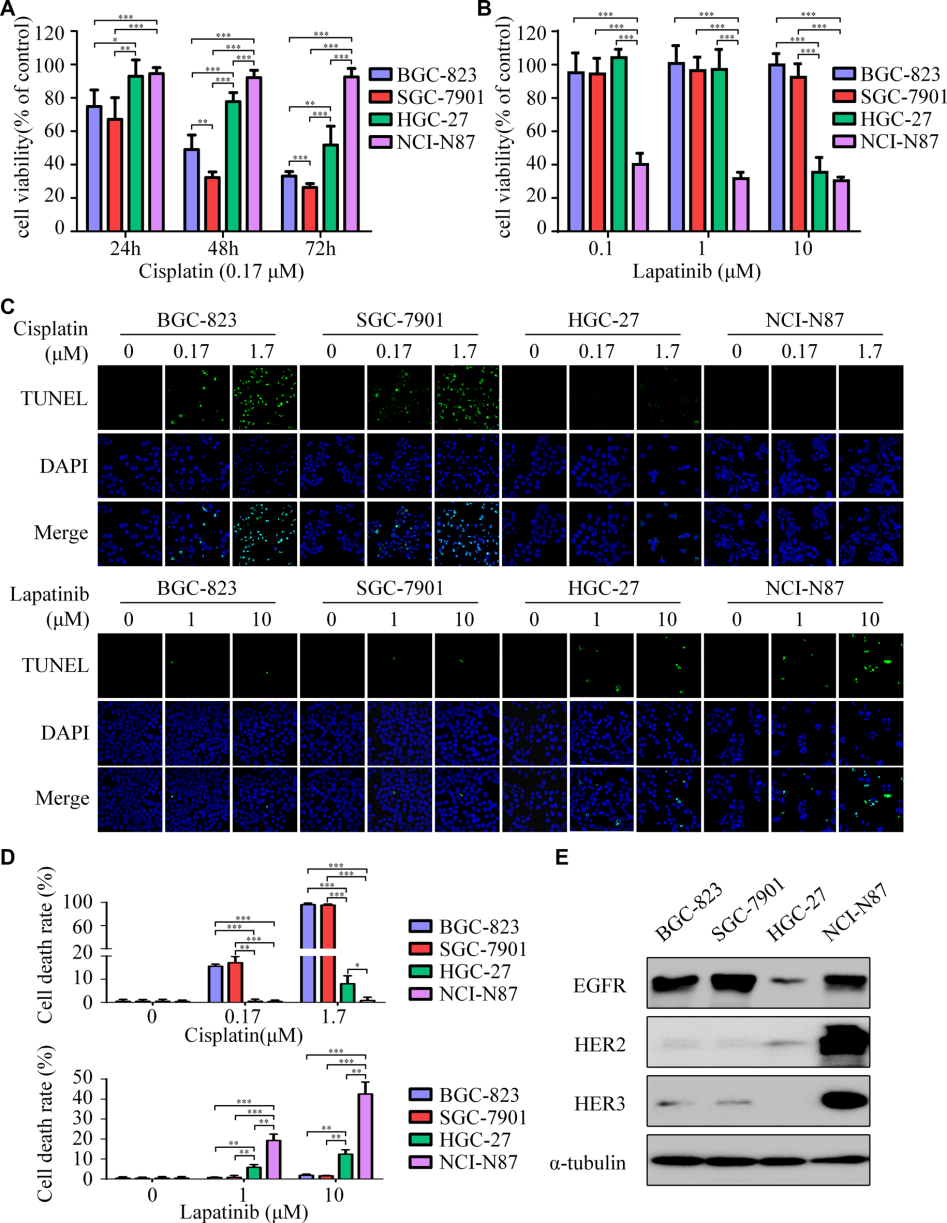
Lapatinib is effective to intrinsic cisplatin-resistant GC cells (**A**) Cell viability was determined by CCK-8 cell-proliferation assay. The gastric cancer (GC) cell lines (BGC-823, SGC-7901, HGC-27, NCI-N87) were exposed to 0.17 μM cisplatin for 24, 48 and 72 h. The percentage of viable cells was shown relative to untreated controls. (**B**) The cell viabilities of four GC cell lines were measured using CCK-8 assay after 48 h exposure to 0, 0.1, 1 and 10 μM of lapatinib. (**C**) Cell death was determined by TUNEL assay (× 1000). Top: The four GC cell lines were treated with 0, 0.17, 1.7 μM cisplatin for 24 h. Bottom: The four GC cell lines were treated with 0, 1, 10 μM lapatinib for 24 h. (**D**) Quantify TUNEL-positive cells of GC cells treated with cisplatin or lapatinib. (**E**) Western blot showing EGFR, HER2 and HER3 protein expressions in the four GC cell lines. The data presented are means ± SD from three independent experiments. ^*^P < 0.05, ^**^P < 0.01, ^***^ *P* < 0.001.

BGC-823 and SGC-7901 were extremely sensitive to cisplatin, whereas HGC-27 was less sensitive and NCI-N87 presented primary resistance to cisplatin (Figure 1A). Interestingly, results indicated that BGC-823 and SGC-7901 displayed resistance to lapatinib. In contrast, NCI-N87 cells were the most responsive to lapatinib in these cell lines, with a half-maximum inhibitory concentration (IC_50_) of 0.09 μM. HGC-27 cells have a moderate IC_50_ of 7.73 μM (Figure 1B and Supplementary Table S1). In addition, significant apoptosis was induced by cisplatin in BGC-823 and SGC-7901, but inconspicuous one in HGC-27 and NCI-N87 (Figure 1C, top). Conversely, lapatinib induced apparent apoptosis in NCI-N87 and HGC-27, other than BGC-823 and SGC-7901 (Figure 1C, bottom).

The NCI-N87 cell line was highly amplified for the *HER2* gene, while BGC-823, SGC-7901, and HGC-27 were *HER2* negative (Supplementary Figure S1). Moreover, the expression of HER2 protein in HGC-27 was slightly higher than those in BGC-823 and SGC-7901 (Figure 1E). Based on these results, we observed that cisplatin-resistant NCI-N87 cells were highly sensitive to lapatinib. In addition, HER2 expression seemed to have a negative correlation with cisplatin, but a positive one with lapatinib. However, EGFR, HER3, and HER4 were not closely correlated with the sensitivity of these drugs among the GC cell lines.

### Overexpression of HER2 increases lapatinib-induced apoptosis in GC cells

**Figure 2:**
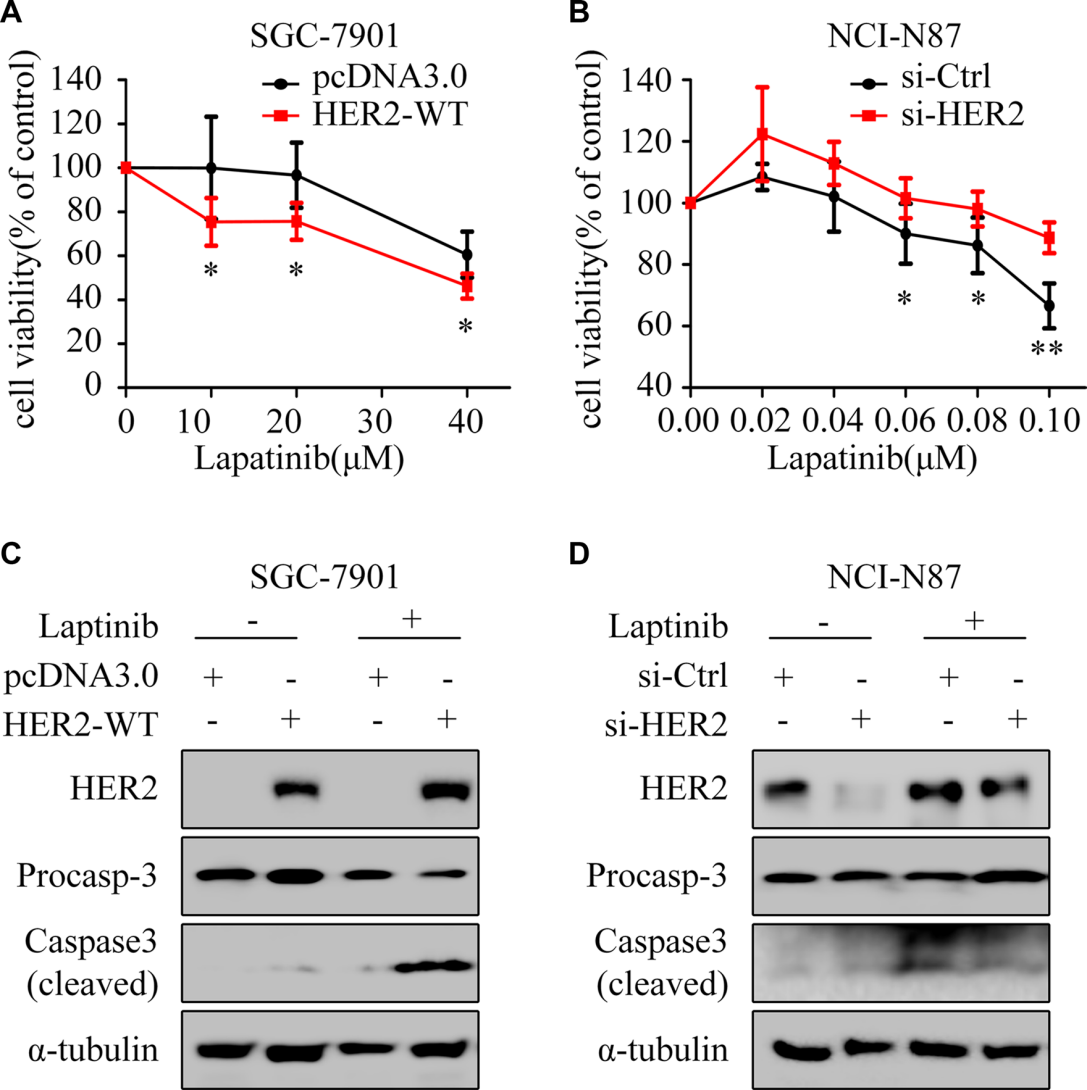
HER2 level contributes to lapatinib sensitivity (**A**) The cell viability was measured by CCK8 assay. SGC-7901 cells were exposed to different concentrations of lapatinib for 24 h after transfection with pcDNA3.0 or HER2-WT plasmid for 48 h. (**B**) NCI-N87 cells transfected with or without HER2 siRNA were treated with varying concentrations of lapatinib for 24 hours. The cell survival rates are expressed as means ± SD from at least three independent experiments. ^*^P < 0.05, ^**^P < 0.01, compared with control group. (**C**) Western blotting for HER2 and Caspase3 with or without HER2 overexpression in the presence or absence of lapatinib (30 μM, 24 h) in SGC-7901 cells. (**D**) Western blotting for HER2 and Caspase3 with or without HER2 knockdown in the presence or absence of lapatinib (1 μM, 24 h) in NCI-N87 cells.

To determine whether HER2 overexpression can rescue the HER2-negative GC cells from lapatinib resistance, HER2-WT plasmid was transfected into SGC-7901 cells. The results showed: overexpression of HER2 enhanced the growth inhibition (Figure 2A) and cleaved caspase3 by lapatinib (Figure 2C). Meanwhile, silencing of HER2 decreased the growth inhibitory effect (Figure 2B) and cleaved caspase3 induced by lapatinib in NCI-N87 (Figure 2D).

### Expression of JWA sensitizes cisplatin-resistant GC cells to lapatinib-triggered apoptosis

**Figure 3:**
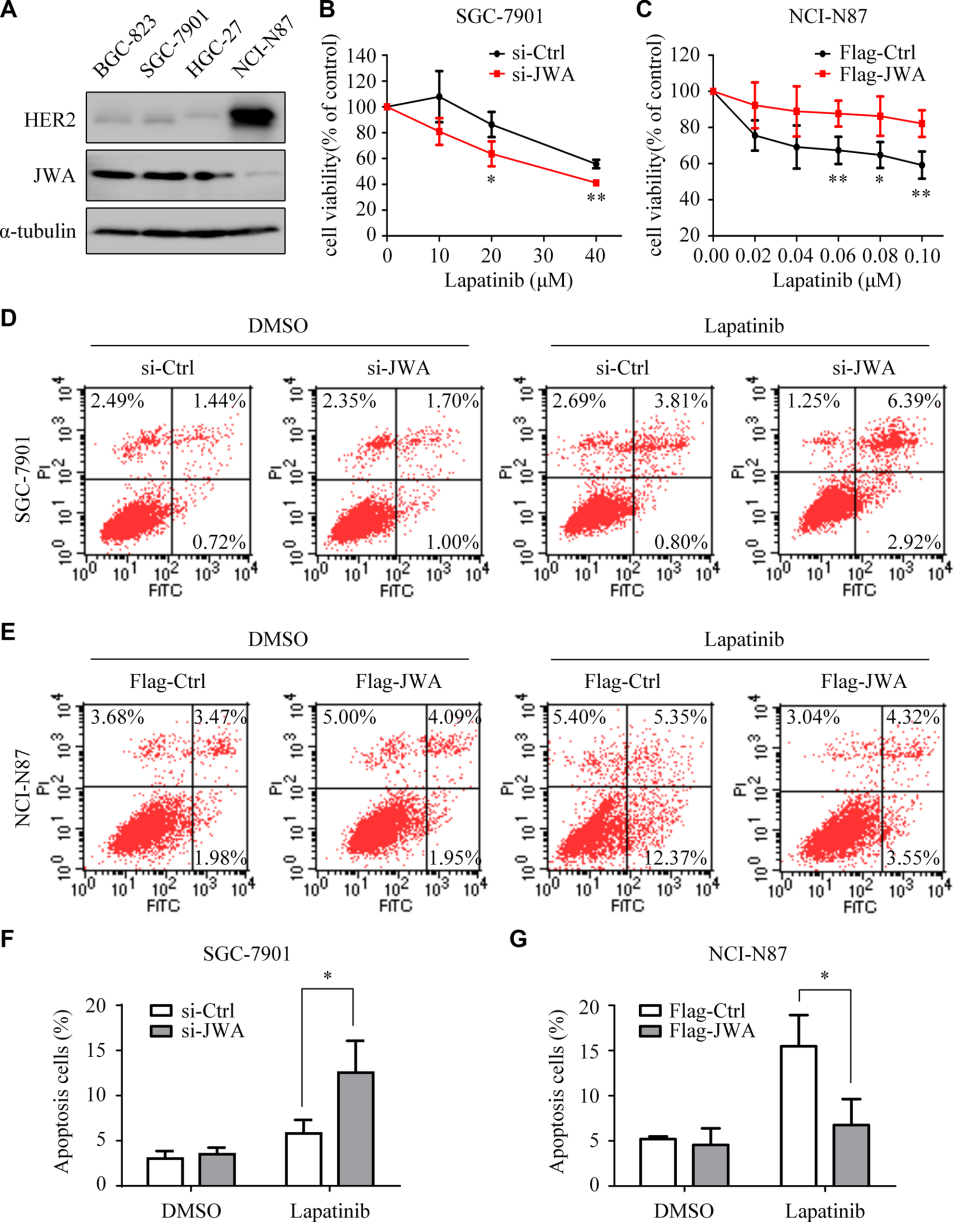
JWA decreases the sensitivity of GC cells to lapatinib (**A**) Expressions of HER2 and JWA were examined in whole-cell lysates by Western blotting. (**B** and **C**) SGC-7901 cells with or without JWA knockdown (B) and NCI-N87 cells with or without JWA overexpression (C) were treated with the indicated doses of lapatinib for 24 h. Cell survival was determined using the CCK8 assay. The cell survival rates are presented as means ± SD from three independent experiments. (**D**) SGC-7901 cells were transfected with si-JWA or its vector for 48 h, followed by incubation with 30 μM lapatinib for 24 h, and then analyzed by flow cytometry. (**E**) NCI-N87 cells were transfected with Flag-JWA or its vector for 48 h, followed by incubation with 1 μM lapatinib for 24 h, and then analyzed by flow cytometry. (**F** and **G**) Quantification of apoptosis in D and E. Columns indicate average of triplicates and bars indicate SD. ^*^P < 0.05, ^**^P < 0.01.

Next, we observed opposite expression patterns of JWA and HER2 in lapatinib sensitive and resistant GC cells (Figure 3A). Lapatinib resistant BGC-823 and SGC-7901 revealed obvious JWA activation. Indeed, transfection of JWA siRNA into SGC-7901 cells significantly restored lapatinib suppression on proliferation (Figure 3B). Through FACS analysis, we found that silencing of JWA increased the apoptosis rate of lapatinib in SGC-7901 (Figure 3D). Conversely, JWA activation distinctly weakened lapatinib inhibition on proliferation (Figure 3C) and reduced the cell apoptosis rate of lapatinib in NCI-N87 cells (Figure 3E).

### JWA promotes lapatinib resistance in GC cells through down-regulation of HER2

**Figure 4:**
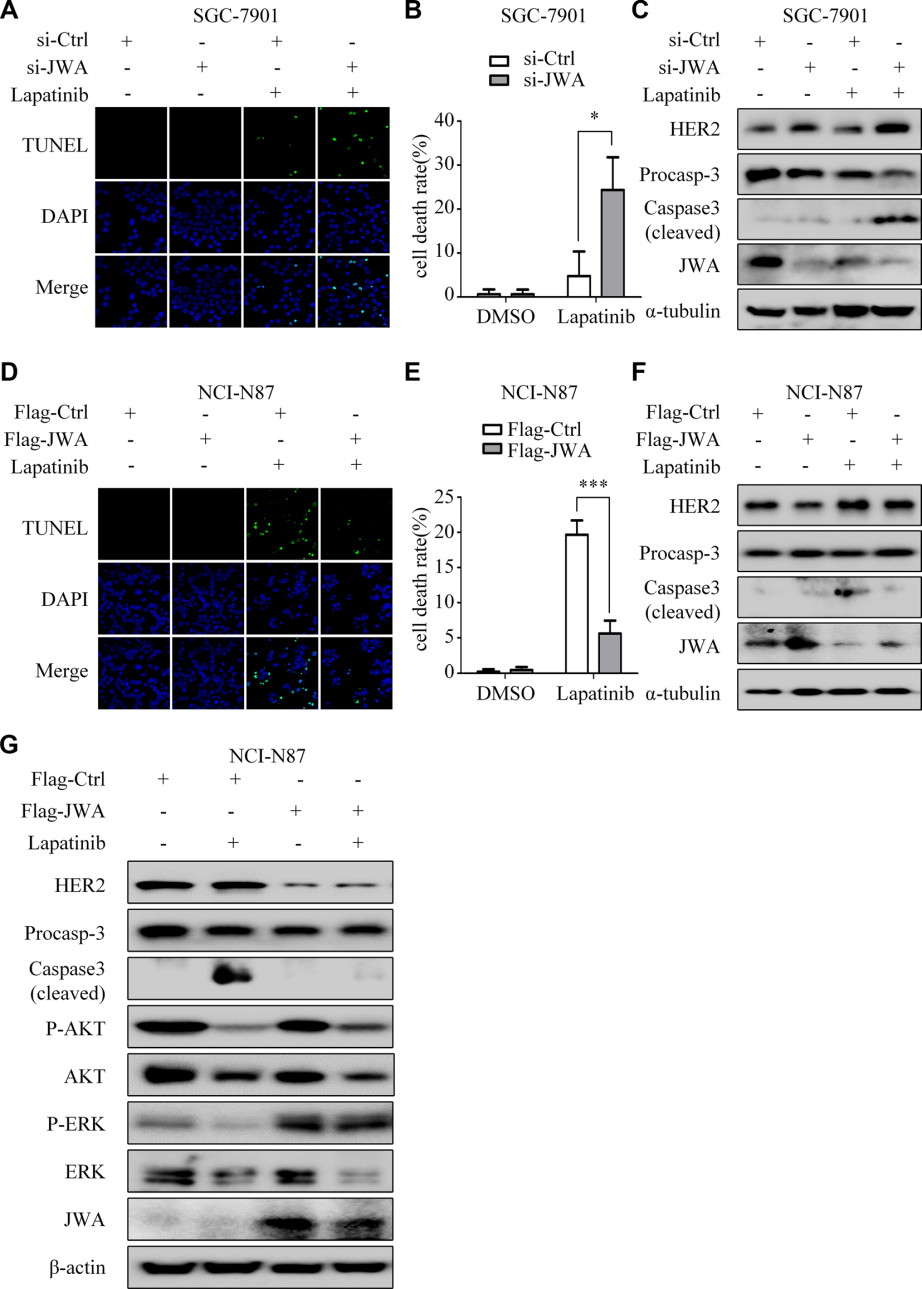
JWA mediates lapatinib resistance by negatively regulating HER2 (**A**) SGC-7901 cells were transfected with JWA siRNA or 48 h, followed by exposure to 30 μM lapatinib for 24 h. The apoptotic rate was determined by the TUNEL assay (×1000). (**B**) Quantification of TUNEL-positive SGC-7901 cells transfected with JWA siRNA. (**C**) SGC-7901 cells were treated with lapatinib as in (A) and whole-cell lysates were collected for detection of target proteins by Western blotting. (**D**) NCI-N87 cells were transfected with Flag-JWA and then treated with 1μM lapatinib for 24 h. The apoptotic rate was determined by the TUNEL assay (× 1000). (**E**) Quantification of TUNEL-positive NCI-N87 cells transfected with Flag-JWA. Columns indicate average of triplicates and bars indicate SD. ^*^P < 0.05, ^***^P < 0.001. (**F**) NCI-N87 cells were treated with lapatinib as in (D) and whole-cell lysates were collected for detection of target proteins by Western blotting. (**G**) Effects of lapatinib on downstream regulatory molecules of HER2. NCI-N87 cells with or without JWA overexpression were cultured in the presence or absence of lapatinib (1 μM, 24 h). Whole-cell lysates were collected for detection of target proteins by Western blotting.

The TUNEL assays indicated that the apoptotic rates induced by lapatinib were significantly increased in JWA silenced SGC-7901 cells (Figure 4A and 4B), but decreased in JWA overexpressed NCI-N87 cells (Figure 4D and 4E). Moreover, deletion of JWA in SGC-7901 cells led to up-regulation of HER2 and lapatinib-induced cleaved caspase-3 (Figure 4C). Conversely, in NCI-N87 cells transfected with Flag-JWA, HER2 expression was down-regulated, and the level of lapatinib-induced cleaved caspase-3 was evidently attenuated (Figure 4F).

As a dual tyrosine kinase inhibitor (TKI) of both HER2 and EGFR, lapatinib interrupts the downstream signaling pathways such as MAPK and AKT [18]. Our results showed that: phosphorylations of AKT and ERK were suppressed by lapatinib both with and without JWA activation, whereas phosphorylation of ERK was slightly inhibited by lapatinib after transfection of Flag-JWA (Figure 4G and Supplementary Figure S2).

### JWA enhances HER2 degradation in GC cells

**Figure 5:**
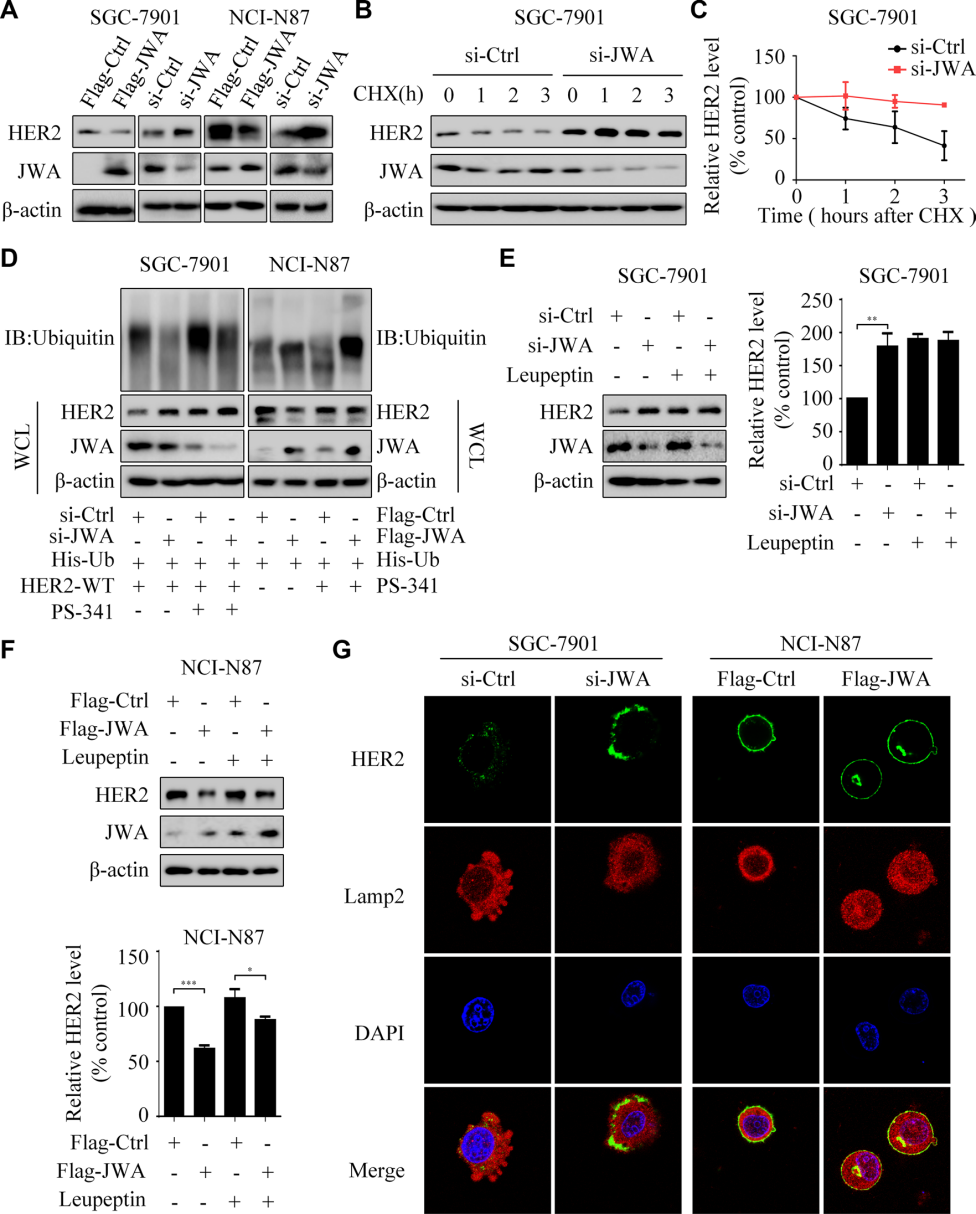
JWA is required for degradation of the HER2 protein (**A**) The SGC-7901 and NCI-N87 cells were transfected with Flag-JWA or JWA siRNA for 48 h. Western blot showed the expressions of HER2 and JWA. (**B** and **C**) SGC-7901 cells were transfected with JWA siRNA for 48 h, followed by treatment with 178 μM of CHX at indicated times. The protein stability of HER2 was assessed by western blotting (B). Quantification of HER2 protein levels (C). (**D**) The SGC-7901 cells were transfected with JWA siRNA, His-Ub and HER2-WT for 48 h, followed by treatment with 50 μM of PS-341 for 6 h. The NCI-N87 cells were transfected with Flag-JWA and His-Ub for 48 h, followed by treatment with 50 μM of PS-341 for 6 h. Western blotting was performed to confirm the levels of HER2 and JWA. Immunoprecipitation was used to show the ubiquitination of HER2. (**E** and **F**) SGC-7901 cells transfected with JWA siRNA or NCI-N87 cells transfected with Flag-JWA plasmid for 24 h, then incubated with or without leupeptin (5 μM) for 20 h. Western blotting was carried out to confirm the level of HER2 and JWA (E. left and F. top). Her2 bands were normalized to β-actin. The data represent means ± SD of triplicate experiments (E. right and F. bottom). ^*^P < 0.05, ^**^P < 0.01, ^***^P < 0.001. (**G**) SGC-7901 were transfected with si-JWA (left) and NCI-N87 were transfected with Flag-JWA (right) for 48 h. Immunofluorescence imaging of HER2 (green), the lysosome marker Lamp2 (red), nucleus labeled as DAPI (blue), the co-localization of the three signals (merge).

We further tested whether the proteasome or lysosome pathway was responsible for the negative regulation between JWA and HER2. Deletion of JWA reduced the degradation of HER2 in SGC-7901 treated with CHX (Figure 5B and 5C). Moreover, when treated with proteasome inhibitor PS-341, the HER2 expression was obviously up-regulated in the SGC-7901 cells transfected with si-Ctrl (Figure 5D left). In addition, PS-341 effectively rescued HER2 expression in the NCI-N87 cells transfected with Flag-JWA (Figure 5D right). Concurrently, ubiquitinated HER2 was increased because PS-341 inhibited its degradation (Figure 5D).

Leupeptin, a lysosome inhibitor, was able to reverse the decreased HER2 triggered by JWA (Figure 5E and 5F). In si-Ctrl transfected SGC-7901 (a HER2 negative cell line), a small extent of HER2 (green) colocalized with cytoplasmic lysosome marker Lamp2 (red). However, when JWA was silenced, the overall HER2 expression distinctly elevated (Figure 5G left). Inversely, in NCI-N87, a HER2 positive cell line, overexpression of JWA caused relocation of HER2 and cytoplasmic lysosomes (Figure 5G right).

### JWA mediates c-Cbl to down-regulate HER2 expression in GC cells

**Figure 6:**
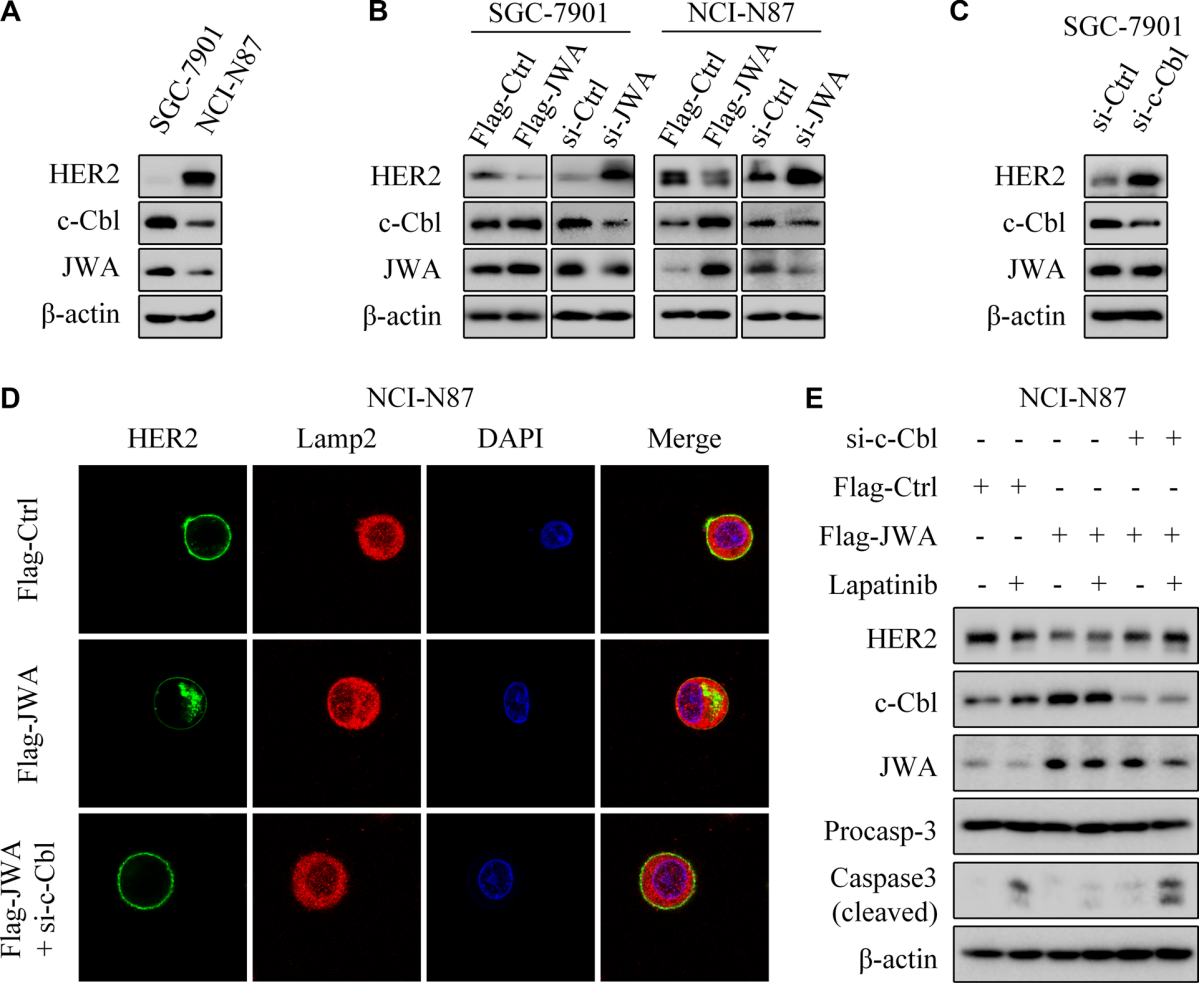
JWA negatively regulates HER2 expression via c-Cbl in GC cells (**A**) Western blotting was used to determine the expressions of HER2, c-Cbl and JWA in SGC-7901 and NCI-N87 cells. (**B**) SGC-7901 and NCI-N87 were transfected with Flag-JWA or JWA siRNA for 48 h, target proteins were determined with Western blot. (**C**) SGC-7901 cells were transfected with c-Cbl siRNA for 48 h, and western blotting showed the expression of target proteins. (**D**) NCI-N87 were transfected with Flag-Ctrl (top), Flag-JWA (middle), or cotransfected with Flag-JWA and c-Cbl siRNA (bottom) for 48 h. Immunofluorescence imaging of HER2 (green), the lysosome marker Lamp2 (red), nucleus labeled as DAPI (blue), the co-localization of the three signals (merge). (**E**) NCI-N87 cells were cotransfected with Flag-JWA and c-Cbl siRNA for 48 h, incubated with or without lapatinib (1 μM) for 24 h. Western blotting was used to determine the expression of target proteins.

C-Cbl and HSP90 have been identified to play significant roles in the regulation of HER2 through post-transcriptional events [19–21]. JWA and c-Cbl presented significant expressions in HER2 negative SGC-7901 cells, other than in HER2-amplified NCI-N87 cells (Figure 6A). We next examined the expressions of c-Cbl and HER2 after transfection with Flag-JWA plasmid or JWA siRNA. It revealed the evident dependence of c-Cbl upon JWA (Figure 6B). Nevertheless, no remarkable difference of HSP90 was observed in these GC cells (Supplementary Figure S3A and S3B). Deletion of c-Cbl up-regulated HER2 expression, but did not affect JWA protein level (Figure 6C).

Intriguingly, JWA-induced perinuclear aggregates of HER2 and partial co-localization of HER2 with Lamp2 were decreased after transfection of c-Cbl siRNA (Figure 6D). Cotransfection with Flag-JWA and si-c-Cbl abrogated the decreased HER2 expression by JWA (Figure 6E). Moreover, the cotransfection almost abolished the apoptotic suppression by JWA under lapatinib treatment.

## DISCUSSION

Here, we demonstrated for the first time that JWA can be a biomarker for lapatinib and reported a novel pathway how JWA regulates HER2 in GC cells. We have provided new evidences supporting that degradation of HER2 and phosphorylation of ERK induced by JWA can be the factors for lapatinib resistance in GC cells. Moreover, we revealed a new mechanism for the significant role of c-Cbl in HER2 degradation enhanced by JWA in GC cells.

Our study showed new evidences that cisplatin-resistant GC cells sensitized to lapatinib. Thus, we hypothesize that lapatinib could be an option for cisplatin-resistant GC patients, improving the curative effect and reducing the adverse effect. Our results also indicated that lapatinib effectively induced apoptosis in HER2-positive GC cells and HER2 expression levels were associated with lapatinib sensitivity, which were consistent with previous studies [22, 23]. The stabilization of HER2 partly explains the differently displayed HER2 status [24]. Given the extensive investigations into HER2 post-translation modification [19, 25], the present study attempted to cast new light on these processes by evaluating HER2 down-regulation and degradation activated by JWA. We noted low expression of JWA in the NCI-N87 cell line which was sensitive to lapatinib. Conversely, over-expression of JWA was observed in GC cell lines resistant to lapatinib. Moreover, the expression of JWA sensitized cisplatin-resistant GC cells to lapatinib-triggered apoptosis. These data indicated that JWA was a considerable factor in determining susceptibility to lapatinib in GC cells.

Tomohiro Shibata et al. reported that silencing of YB-1 specifically down-regulates HER2 expression and induces lapatinib resistance in GC cells [26]. The data presented here indicated that sensitivity of lapatinib altered by JWA was dependent on HER2 expression. HER2-independent activation of PI3K-AKT and ERK pathways have been proposed as mechanisms of lapatinib resistance [27, 28]. Our study also demonstrated that JWA especially promoted ERK phosphorylation with or without treatment of lapatinib. Rescuing HER2-positive GC cells from lapatinib inhibition was partially ascribed to activation of ERK by JWA [29]. Further research is needed to understand the mechanism. These results suggested that JWA was a considerable factor in determining GC cells susceptibility to lapatinib-induced apoptosis via regulation of HER2 expression and activation of MAPK/ERK pathway.

**Figure 7:**
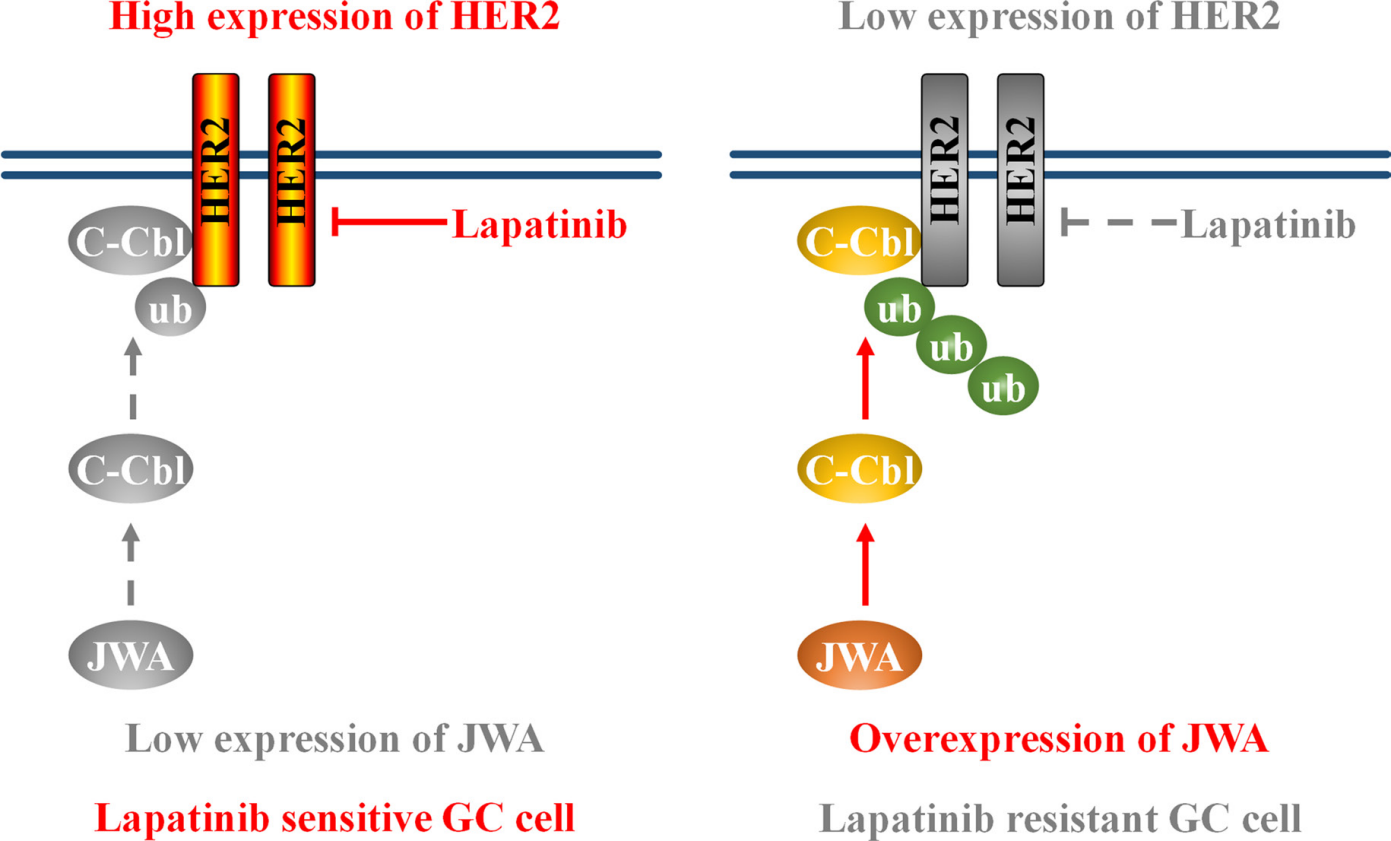
Schematic model of the JWA/c-Cbl/HER2 pathway's role in lapatinib resistance Left: low expression of JWA contributes to HER2 stabilization and lapatinib sensitivity. Right: JWA overexpression promotes the E3 ubiquitin ligase c-Cbl, leading to an increase in Her2 polyubiquitination. This ultimately results in a decrease in Her2 protein levels and confers lapatinib resistance.

More importantly, we demonstrated for the first time that JWA might act through c-Cbl to induce HER2 degradation in lapatinib sensitive and resistant GC cells. The results revealed that JWA overexpression increased c-Cbl expression, which resulted in HER2 receptor degradation. Previous studies showed that c-Cbl belongs to ubiquitin ligase E3 family and functions as a negative regulator for HER2 via a c-Cbl dependent endocytosis, polyubiquitination and degradation pathway [24, 30]. Our data revealed that JWA was able to accelerate HER2 degradation and to increase the colocalized portion of HER2 and Lamp2 in cytoplasm. However, deletion of c-Cbl attenuated JWA-induced co-localization between HER2 and Lamp2. Furthermore, knockdown of c-Cbl rescued JWA-induced HER2 down-regulation and lapatinib resistance. These data suggested that the JWA/c-Cbl/HER2 pathway played a predominant role in lapatinib resistance (Figure 7).

Our previous study reported that JWA regulates DNA damage-apoptosis induced by cisplatin and JWA may be a valuable target for reversal of cisplatin resistance in human GC [17]. In this study, we observed that low expression of JWA sensitizes cisplatin-resistant GC cells to lapatinib-induced apoptosis. Therefore, in GC patients, low expression of JWA can be utilized as a biomarker for lapatinib treatment, while higher expression of JWA can be utilized to identify the GC patients benefiting from cisplatin treatment. Further clinical studies are needed to examine the biomarkers.

In conclusion, we report for the first time that JWA promotes lapatinib resistance in GC cells via down-regulation of the HER2 protein expression and meanwhile through increasing ERK phosphorylation. Our data also suggested that low level of JWA can be utilized as a predictive marker for lapatinib sensitivity and identifying the GC patients that may benefit from lapatinib or cisplatin treatment. Furthermore, we postulate that the JWA/c-Cbl/HER2 axis could contribute to the further development of personalized therapeutics by HER2-targeted drugs against human GC. Prospective clinical studies are warranted in order to further evaluate these biomarkers.

## MATERIALS AND METHODS

### Cell lines and culture

Human GC cell lines SGC-7901, BGC-823 and HGC-27 were purchased from the Type Culture Collection of Chinese Academy of Sciences (Shanghai, China). NCI-N87 cells were purchased from the American Type Culture Collection (ATCC, USA). Cells were cultured in RPMI 1640 medium (Gibco, USA) supplemented with 10% fetal bovine serum (FBS; Gibco, USA), 100 U/ml penicillin and 100 μg/ml streptomycin (Gibco, USA). The cells were incubated in a humidified atmosphere under 5% CO2 at 37°C.

### Drug preparations and reagents

Lapatinib (Selleck Chemicals, USA) was dissolved in 100% dimethyl sulfoxide (DMSO; Sigma-Aldrich, USA) and then diluted with culture medium to the desired concentration. DMSO added in treatment groups was equal to the control ones with a final DMSO concentration < 0.2% (v/v). Cisplatin and cycloheximide (CHX) was obtained from Sigma-Aldrich (St. Louis, MO, USA). PS-341 was purchased from Selleck Chemicals (USA). Leupeptin was bought from Amresco (USA).

### Plasmids and transfections

The Flag-Ctrl and Flag-JWA plasmids have been described previously. The HER2 Wild Type (HER2-WT) construct was obtained from Professor Mien-Chie Hung (The University of Texas MD Anderson Cancer Center, USA). The siRNAs specific for JWA, HER2 and c-Cbl were synthesized (RiboBio, Guangzhou, China). The plasmid DNA or siRNA was transiently transfected into cells with Lipofectamine 3000 (Invitrogen, USA) according to the manufacturer's protocol.

### Cytotoxicity assay

The cells were seeded at 5,000 cells per well in 96-well plates and incubated overnight. After treatment with various concentrations of lapatinib or cisplatin for a certain time, the cell viability was determined using Cell Counting Kit-8 (CCK8, Dojindo, Japan) according to the manufacturer's instructions. IC_50_ was evaluated through non-linear regression model in GraphPad Prism Software (La Jolla, CA, USA). The cell survival rates are expressed as means ± SD from at least three independent experiments.

### Apoptosis assay

NCI-N87 cells or SGC-7901 cells were seeded in 6-well plates and transiently transfected with plasmid DNA or siRNA. Two days after transfection, the cells were treated with certain concentrations of lapatinib for indicated time points. Both floating and adherent cells were harvested and stained with Annexin V and Propidium iodide (Dojindo, Kumamoto, Japan) and further analyzed with a flow cytometry (FACScan, BD Biosciences, USA) equipped with a Cell Quest software (BD Biosciences, USA). Apoptosis was also determined using the TUNEL apoptotic cell detection kit (Roche, Basel, Switzerland), according to the manufacturer's instructions.

### Western blotting and antibodies

Western blotting was performed as previously described [31]. The antibodies used were as follows: monoclonal anti-JWA (1 : 500, contract produced by AbMax, Beijing, China); monoclonal anti-α-tubulin, anti-β-actin (loading control) (1 : 2000, Beyotime, Haimen, Jiangsu, China); monoclonal anti-P-ERK, anti-ERK, anti-P-AKT(473), anti-AKT, anti-Caspase 3, anti-EGFR, anti-HER3 (1 : 1000, Cell Signaling Technology, Danvers, MA, USA); monoclonal anti-Ub (1 : 500) and polyclonal anti-HER2 (1 : 1000) (Santa Cruz, Dallas, TX, USA); monoclonal anti-c-Cbl (1 : 2000), anti-Lamp2 and anti-HER2 (1 : 250, Abcam, USA).

### Coimmunoprecipitation

Coimmunoprecipitations were performed as previously described [31]. Briefly, the cells were cultured in 6-cm plates and transiently transfected with certain plasmids or siRNA. Two days after transfection, the cells were treated with 50 μM of PS-341 for 6 h. The cell lysates were subjected to immunoprecipitation by incubating with anti-HER2-beads (protein A/G agarose beads, Santa Cruz, USA) overnight at 4°C. Immune complexes were analyzed by western blotting with specific antibodies.

### Immunofluorescence

Cells seeded in 35-mm glass-bottom dishes were cultured overnight, washed with PBS and fixed with 4% PFA for 10 min at room temperature. After cell permeabilization in 0.5% Triton X-100 for 10 min, cells were blocked with 10% normal goat serum for 60 min at room temperature, then incubated with primary antibody (4% serum dilution) overnight. Secondary antibodies were goat anti-rabbit Alexa Fluor 488 and anti-rat Cy3 (Beyotime, China). Cells were incubated with secondary antibody for 1 h. After washing with PBS, cells were stained with VECTASHIELD Mounting Medium for Fluorescence with DAPI (Vector Laboratories, CA) and left in dark before fluorescence microscopic analysis. The confocal images of cells were sequentially acquired with Zeiss AIM software on a Zeiss LSM 700 confocal microscope system (Carl Zeiss Jena, Oberkochen, Germany).

### Quantitative real-time RT-PCR assay

Total RNA was extracted from cell cultures by using the Trizol reagent (Gibco, USA) according to the manufacturer's instructions. Approximately 1 μg of RNA was used for the reverse transcription reaction with OligodT (18T) (Life Technologies). The cDNA was amplified with the following primers: 5′-GCCGGTGCTGAGTATGTC-3′ (forward) and 5′-CTTCTGGGTGGCAGTGAT-3′ (reverse) for GAPDH; 5′-TGTGACTGCCTGTCCCTACAA-3′ (forward) and 5′-CCAGACCATAGCACACTCGG-3′ (reverse) for HER2; 5′-GGAGGAGTCATTGTGGTGC-3′ (forward) and 5′-GAAGTCTCAGGGATGCGTG-3′ (reverse) for JWA. The following thermal cycling conditions were used: denaturation at 94°C for 5 min followed by 36 cycles of denaturation at 94°C for 35 s, annealing at 56°C for 30 s and extension at 72°C for 35 s.

### Statistical analysis

Data are expressed as the means ± SD. The statistical significance of the differences between the cell lines were analyzed by the parametric unpaired Student's *t* test. Differences were considered statistically significant when *P* < 0.05.

## SUPPLEMENTARY MATERIALS FIGURES AND TABLES


