# Supplementary material for: JWA down-regulates HER2 expression via c-Cbl and induces lapatinib resistance in human gastric cancer cells
Source: Oncotarget. 2016 Sep 30;7(44):71790–801. doi: 10.18632/oncotarget.12374 (PMC5342123; doi:10.18632/oncotarget.12374)
Supplement: Supplementary file 1 [file oncotarget-07-71790-s001.pdf]

JWA down-regulates HER2 expression via c-Cbl and induces lapatinib resistance in human gastric cancer cells

Supplementary Materials

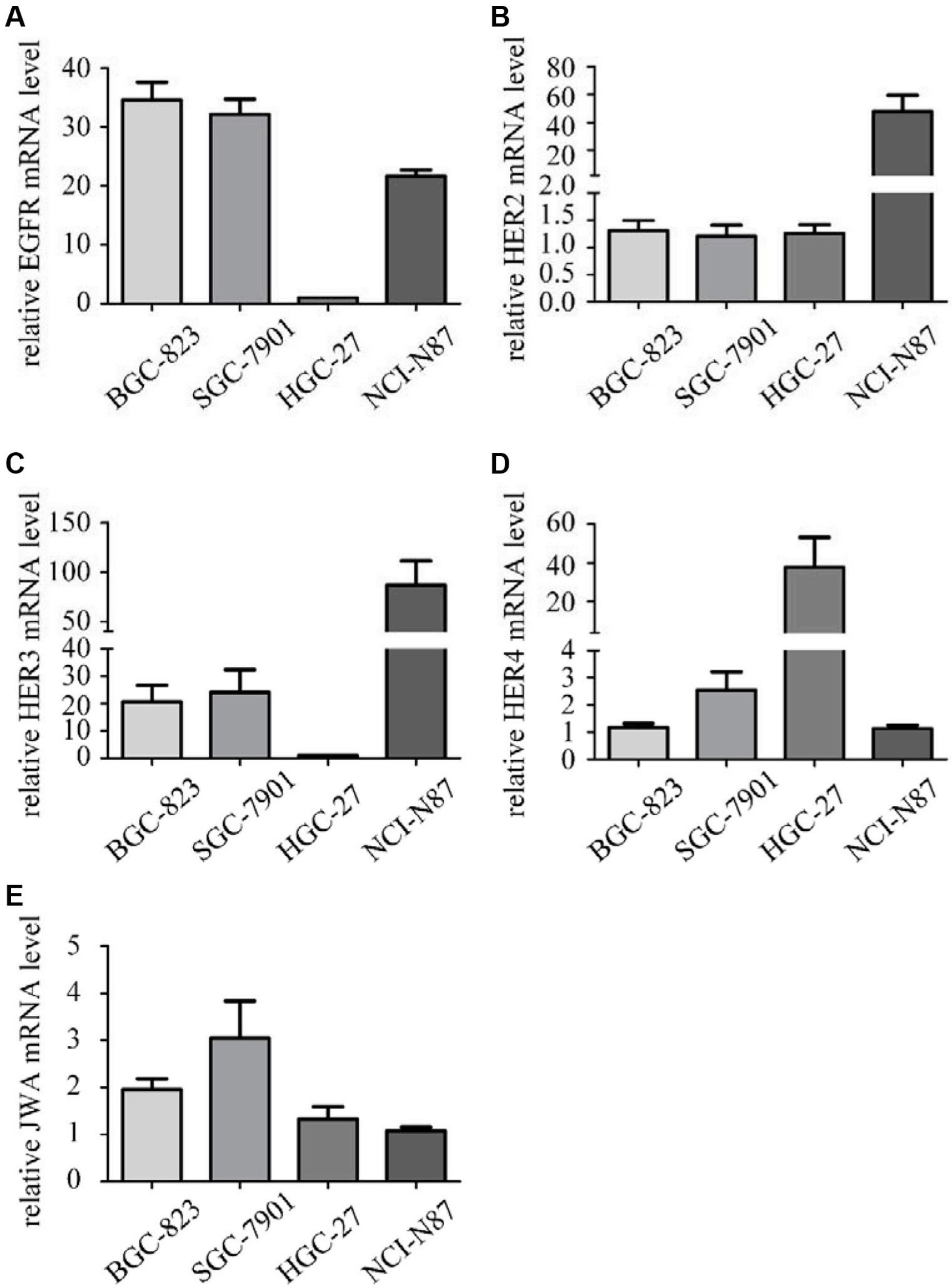

Supplementary Figure S1: Additional data supporting Figure 1E. The mRNA levels of HER family (**A–D**) and JWA (**E**) in BGC-823, SGC-7901, HGC-27 and NCI-N87.

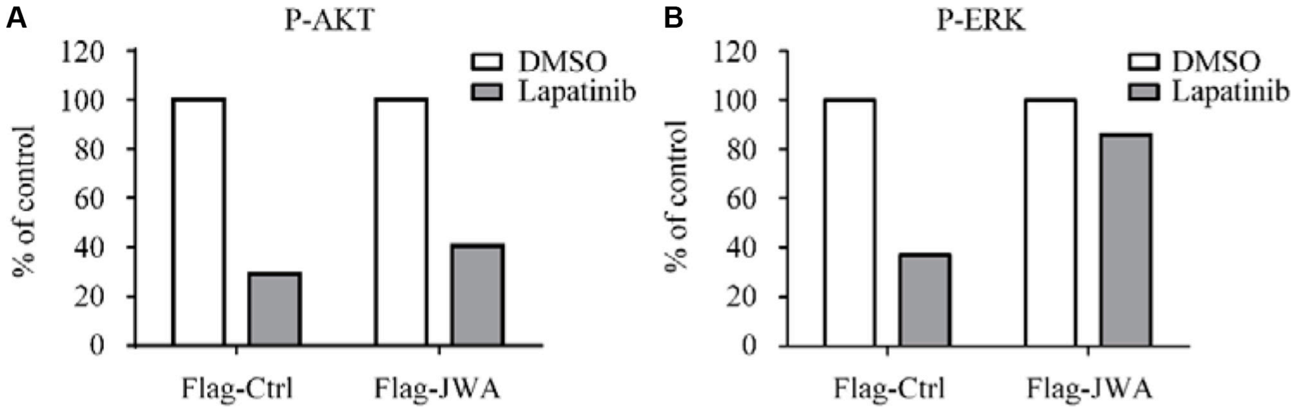

Supplementary Figure S2: Additional data supporting Figure 4G. Band density measurements of P-AKT and P-ERK. (**A**–**B**) The effects of lapatinib on AKT and ERK phosphorylations in figure 4G were quantitative analyzed. Phosphorylations of AKT and ERK were separately normalized by P-AKT and P-ERK protein levels in the absence of the drug.

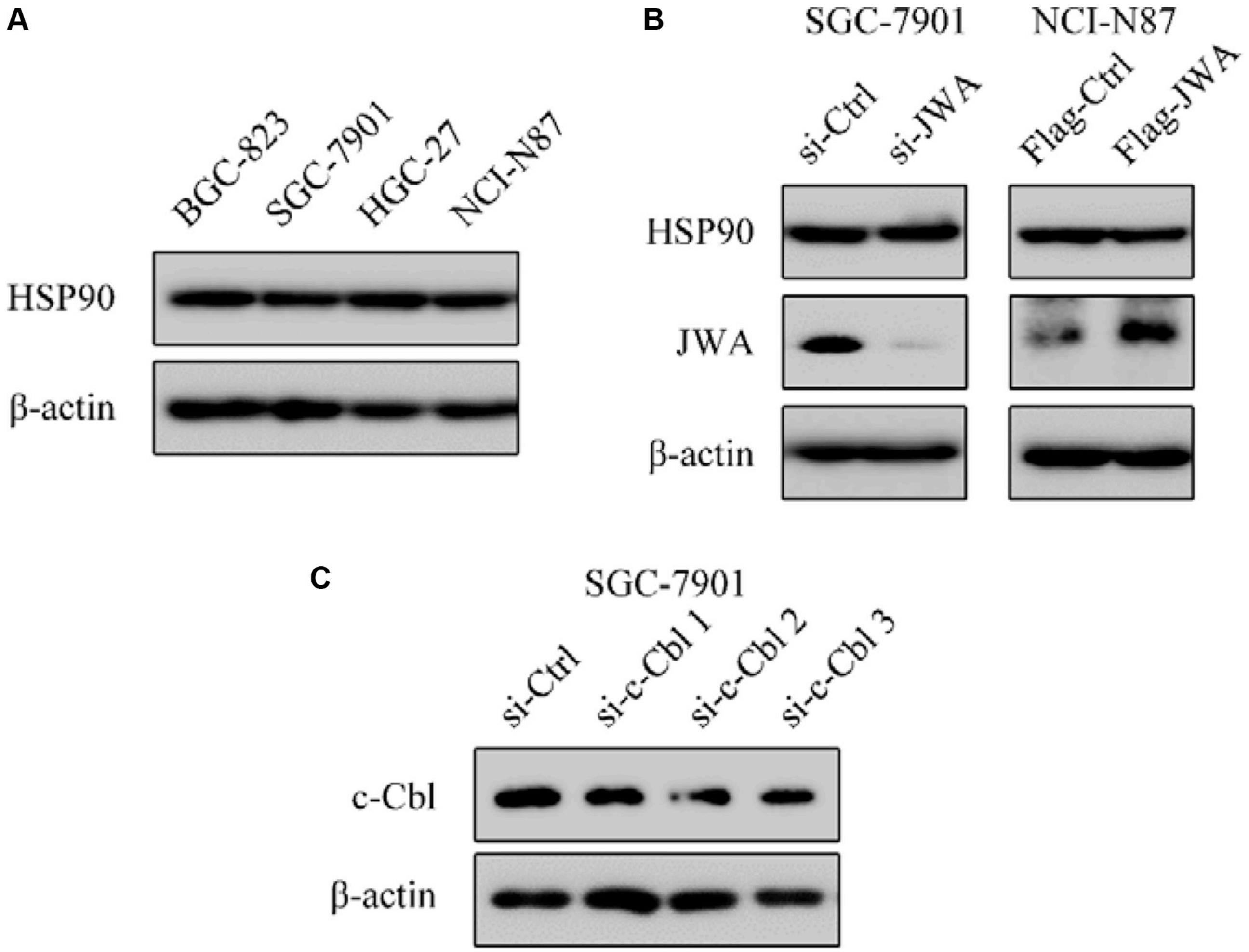

Supplementary Figure S3: Additional data supporting Figure 6. (**A**) Western blotting was used to determine the expression of HSP90 in BGC-823, SGC-7901, HGC-27, NCI-N87 cell lines. (**B**) SGC-7901 cells were transfected with JWA siRNA and NCI-N87 cells were transfected with Flag-JWA for 48 h. Then the protein expression of HSP90 and JWA were measured with Western blot. (**C**) Western blotting was carried out to confirm the effects of the three c-Cbl siRNAs.

Supplementary Table S1: Lapatinib IC_{50} (μM)

Cell line	IC_{50}
BGC-823	67.62
SGC-7901	56.09
HGC-27	7.73
NCI-N87	0.09

NOTE: The IC_{50} values of lapatinib determined using a CCK8 assay as described in Materials and Methods are shown.
